# Supplementary material for: Blood-Derived Exosomal hTERT mRNA in Patients with Lung Cancer: Characterization and Correlation with Response to Therapy
Source: Biomedicines. 2023 Jun 16;11(6):1730. doi: 10.3390/biomedicines11061730 (PMC10295971; doi:10.3390/biomedicines11061730)
Supplement: Supplementary file 1 [file biomedicines-11-01730-s001.zip › biomedicines-2111018-supplementary.pdf]

Supplementary Materials:

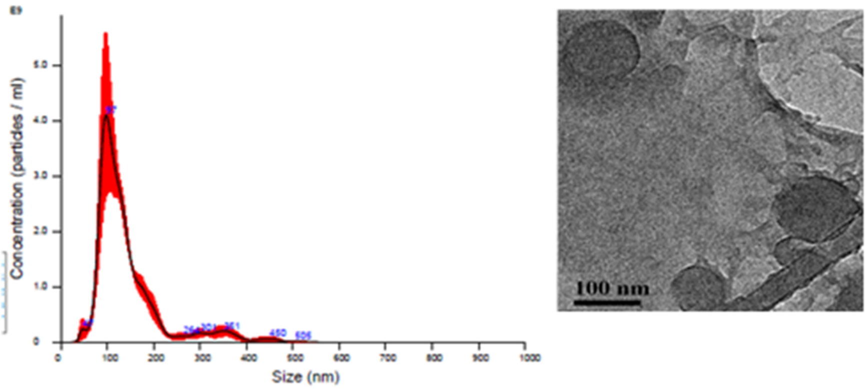

Figure S1. Characterization of the isolated exosomes. Left panel- NanoSight tracking analysis of the isolated extravesicles size and concentration. Right panel- electron microscopy image of the extravesicles size and shape.

Table S1. Patients' characteristics and the levels of exosomal hTERT mRNA.

RQ1	RQ2	RQ3	RQ4	type	staging	response
15.91	0.16	0.29	0.91	NSCLC	IIIb	PR/CR
11.89	0.04	0.04		NSCLC	IV	PR
10.78	0.25			NSCLC	IV	PR
5.02	4.05			SCLC	IV	PR
4.59	0.06	0.00		SCLC	IV	PR
3.72	0.79	2.97	1.08	NSCLC	IIIa	PD
3.69	3.22	3.14		NSCLC	IV	PR
3.63	4.65	0.11		NSCLC	IV	SD
3.55	0.56	0.40	1.39	NSCLC	IV	PD
2.88	0.06			NSCLC	IV	PR
2.55	2.5	2.41		NSCLC	IV	PD
2.03	0.56	0.78	1.59	NSCLC	IV	PR-->SD
1.91	0.96	0.40		NSCLC	IIIa	PR
1.83	1.8			SCLC	IV	PR
1.52	0.18	4.02		SCLC	IV	CR
1.46	0.39	0.52		SCLC	IV	PR
1.39	2.69	1.28	0	NSCLC	IV	SD
1.35	1.4			SCLC	IV	PD
1.34	0.11			NSCLC	IV	PD
1.18	0.12	3.70		NSCLC	IV	PR-->PD
0.68	1.11			NSCLC	IV	PD
0.42	0.04	0.25	0	NSCLC	IV	PR-->SD-->PD
0.40	1.05	0.33		NSCLC	IV	PD
0.38	0.33			NSCLC	IV	PR
0.33	1.09	4.65		NSCLC	IV	PD
0.33	0.24	0.14		NSCLC	IV	CR
0.33	0.29	0.22	0.981	NSCLC	IV	PR
0.30	0.29			NSCLC	IIIb	PD
0.27	0.16			NSCLC	IIIb	PR
0.23		0.35	0.007	NSCLC	IV	PR
0.19	3.10			NSCLC	IIIb	PR
0.16	0.29	0.37	1.66	NSCLC+LCNET	IV	PR
0.15	0.10			NSCLC	IV	PR
0.15		0.60		NSCLC	IV	PR
0.14	0.17	0.16	0.85	NSCLC	IIIb	PR
0.08	0.05	0.00		NSCLC sarcomatoid	IV	PR
0.07	0.00			SCLC	IIIb	SD
0.06	0.62			NSCLC	IV	PD
0.04	0.01	0.63		NSCLC	IV	PR
0.02	0.02	0		NSCLC	IV	PR
0.01	0.00			SCLC	IV	PR
0.00	0.00			SCLC	IV	PR
0.00	0.00	0		NSCLC	IV	PR
